# Characteristics of Highly Sensitive Hydrogen Sensor Based on Pt-WO_3_/Si Microring Resonator

**DOI:** 10.3390/s20010096

**Published:** 2019-12-23

**Authors:** Sosuke Matsuura, Naoki Yamasaku, Yoshiaki Nishijima, Shinji Okazaki, Taro Arakawa

**Affiliations:** Graduate School of Engineering, Yokohama National University, Yokohama 240-8501, Japan; matsuura-sosuke-yf@ynu.jp (S.M.); 0bp66759325232c@ezweb.ne.jp (N.Y.); nishijima-yoshiaki-sp@ynu.ac.jp (Y.N.); okazaki-shinji-yp@ynu.ac.jp (S.O.)

**Keywords:** hydrogen, WO_3_, microring resonator, gas sensor, gas detector

## Abstract

Hydrogen gas has attracted attention as a new energy carrier, and simple but highly sensitive hydrogen sensors are required. We fabricated an optical hydrogen sensor based on a silicon microring resonator (MRR) with tungsten oxide (WO_3_) using a complementary metal-oxide-semiconductor (CMOS)-compatible process for the MRR and a sol-gel method for the WO_3_ layer and investigated its sensing characteristics at device temperatures of 5, 20, and 30 °C. At each temperature, a hydrogen concentration of as low as 0.1 vol% was successfully detected. The gas sensitivity increased with decreasing temperature. The dependence of the sensitivity on the device temperature can be attributed to the thickness of tungsten bronze (H_x_WO_3_) formed by WO_3_ during exposure to hydrogen gas. In addition, a hydrogen gas sensor based on a silicon-MRR-enhanced Mach–Zehnder interferometer (MRR-MZI) is proposed and its significantly high sensing ability using improved changes in the transmittance of light is theoretically discussed.

## 1. Introduction

Recently, hydrogen gas has attracted attention as a new energy carrier. Although hydrogen has an ignition temperature of as high as 585 °C in air, it is flammable, its ignition energy is as small as 0.002 mJ, and its explosive range is from 4.0 to 75 vol%. Therefore, the development of highly sensitive hydrogen gas sensors and their systems capable of detecting low concentrations of hydrogen gas below the lower explosive limit is required [1]. Several papers have been comprehensively reviewed on various types of hydrogen sensor devices [2,3]. Metal-oxide-semiconductor [4] and electrochemical [5,6] sensors have been practically used and showed good performances, and hydrogen gas with a wide range of concentrations between 5 ppm and 100% has been detected. On the other hand, there are still many technical challenges left, such as the reduction of electrical power consumption for device heating, risk mitigation against accidental electric discharge by short-circuit of sensor electrodes, and improvement in durability for easy maintenance, and long-term usage. To solve these problems, room-temperature-operated sensors using functional nanocomposites, for example, have been reported [7,8,9,10,11,12,13]. Hydrogen concentrations of as low as 100 ppm have been detected at room temperature using resistance-type hydrogen sensors [8,11].

Optical sensors including fiber-optic gas sensors are also good candidates for hydrogen gas and have many advantages over conventional sensors [14]. They can provide good reliability because of their immunity to electromagnetic interferences and their explosion-proof structures that have no electrical contacts. Optical hydrogen sensors using optical fibers and nanostructures have been developed [15,16,17,18,19], and hydrogen gas with a concentration as low as 0.1 vol% has been detected [2]. On the other hand, optical gas sensors based on optical waveguides such as microring resonators (MRR) have also been developed for gas sensing [20,21,22,23,24,25,26,27,28,29,30]. In particular, an Si-MRR has a simple structure and a compact footprint and is promising for large scale integrated optical circuits. The Si-MRRs have been applied to sensors for various gases [20,24,26,27,29,30]. However, most of the MRR-based sensors are for ammonia, CO, CO_2_, and volatile organic compound gases. There are few reports on experimental hydrogen gas sensing [20,30].

In this study, we have investigated a highly sensitive silicon-on-insulator (SOI)-MRR-based hydrogen sensor with Pt-doped tungsten oxide (WO_3_) with high sensitivity and rapid response. WO_3_ tis a suitable material for hydrogen gas sensing [19]. When hydrogen gas is exposed to a WO_3_ film, WO_3_ reacts with hydrogen atoms to form tungsten bronze (H_x_WO_3_). As a result, the optical properties of the film, such as the absorption coefficient and refractive index, change, and transmittance of light also changes. Many of the optical hydrogen gas sensors with WO_3_ use these effects [15,16,19,28]. On the other hand, the proposed silicon (Si)-MRR-based hydrogen sensor uses the exothermic reaction in the WO_3_ film. A WO_3_/Si-MRR hydrogen sensor has already been proposed and hydrogen gas with a concentration of 1 vol% was successfully detected [20]. However, its detailed sensing characteristics, such as response times, have not been discussed. We have also obtained sensing characteristics for pure (100 vol%) hydrogen using a WO_3_/Si-MRR sensor [30]. However, its response to hydrogen was very slow (typically approximately 20 min), and the sensing characteristics for lower hydrogen concentrations have never been investigated. In this paper, we show that it is possible for the MRR sensor to detect hydrogen with a concentration of as low as 0.1 vol% and a response time as short as 10 s. The MRR sensor has realized the highest sensitivity and the fastest response at room temperature as an optical hydrogen sensor based on optical waveguides to the best of our knowledge.

## 2. Structure and Operation Principle of Sensor

### 2.1. Structure

**Figure 1:**
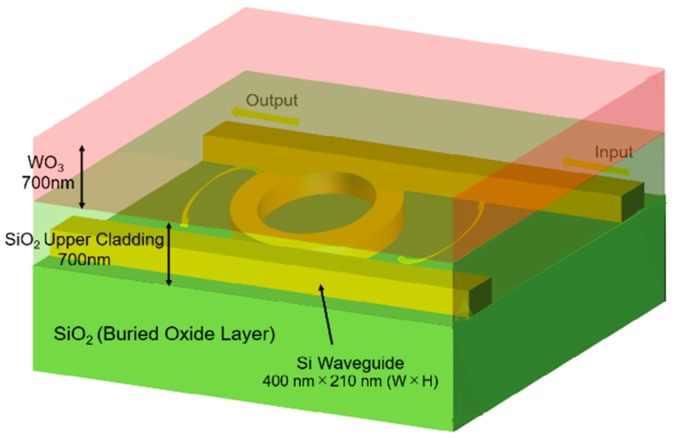
Schematic view of the proposed Pt-WO_3_/Si microring resonator (MRR)-based H_2_ gas sensor.

Figure 1 shows a schematic view of the proposed Pt-WO_3_/Si-MRR-based H_2_ gas sensor. The racetrack-type Si-MRR is coupled with two busline waveguides through directional couplers. The optical power coupling efficiency *K* is controlled by the length of the directional coupler. The waveguides are Si wires with a height of 210 nm and a width of 400 nm. The Si waveguides are surrounded by SiO_2_ cladding layers. The upper cladding layer of the waveguide of the MRR is composed of Pt-WO_3_/SiO_2_ layers. The thicknesses of the Pt-WO_3_ and SiO_2_ upper cladding layers are both 700 nm. The MRR is thermo-optic-driven and senses a change in temperature. When the temperature in the MRR changes, the equivalent refractive index of the waveguide also changes, leading to a shift in the resonant wavelength of the MRR. The device parameters of the sensor are summarized in Table 1.

### 2.2. Principle of Sensing

The resonant wavelength of an MRR is given by

(1) $$\lambda_{0}=\frac{L\cdot n_{eq}}{N},$$

where *N* is the resonance order. When *n*_eq_ changes, *λ*_0_ shifts in the longer/shorter wavelength direction. WO_3_ is known as a gasochromatic material; when it absorbs hydrogen atoms, it forms tungsten bronze (H_x_WO_3_) with the reaction given by

(2) $$(x/2)H_{2}+{\mathrm{WO}}_{3}\to H_{x}{\mathrm{WO}}_{3}.$$

Upon reaction with oxygen, it returns to WO_3_ with the reaction

(3) $$H_{x}{\mathrm{WO}}_{3}+(x/4)O_{2}\to (x/2)H_{2}O+{\mathrm{WO}}_{3}.$$

To induce the above reactions at room temperature, Pt is doped into WO_3_ as a catalyst. Reactions (2) and (3) are exothermic. Therefore, when hydrogen atoms are absorbed into the WO_3_ layer of the upper cladding layers, the temperature of the Si waveguide of the MRR and *n*_eq_ increase, leading to a redshift in resonant wavelength owing to the thermo-optic (TO) effect of Si. The measured temperature coefficient of the resonant wavelength, d*λ*_0_/d*T*, was approximately 57 pm/°C, which is slightly smaller than the theoretical value (72.2 pm/°C) calculated using the TO coefficient dΔ*n*/d*T* of Si (1.62 × 10^−4^). Owing to reaction (2), the refractive index and extinction coefficient of the cladding layer also change, as shown in Table 2.

**Figure 2:**
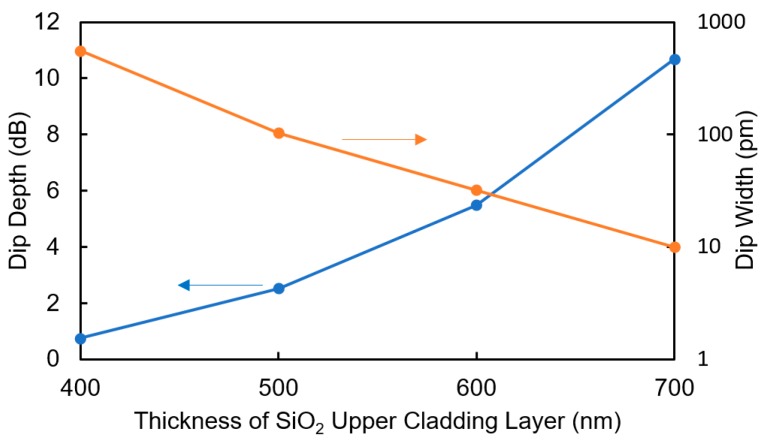
Calculated dip depth and width at 0.1 dB from the bottom of the dip for the MRR with *L*_MRR_ = 69.3 μm and *K* = 0.2 as a function of the thickness of the upper SiO_2_ layer.

In the proposed sensor, only the TO effect induces the shift in the resonant wavelength, and it is preferable that the effects of the changes in the refractive index and extinction coefficient should be removed. Therefore, the thickness of the SiO_2_ upper cladding layer between the WO_3_ layer and the Si waveguide is important. The thicker the SiO_2_ layer, the lower the propagation loss in the MRR, which increases the depth and sharpness of the dip. As a result, the dip can be observed more clearly but the sensitivity decreases. Figure 2 shows the calculated dip depth and width at 0.1 dB from the bottom of the dip for the MRR with *L*_MRR_ = 69.3 μm and *K* = 0.2 as a function of the thickness of the upper SiO_2_ layer. When the thickness of the SiO_2_ layer is 700 nm, a sufficient dip depth of as large as 10 dB is obtained. Moreover, if we assume that the dip depth at 0.2 dB from the bottom of the dip is required to read the wavelength at the bottom of the dip with an accuracy higher than 10 pm, a SiO_2_ thickness of more than 700 nm is required. Therefore, the thickness of the SiO_2_ layer in the device was designed to be 700 nm in the experiment.

The sensitivity depends on the thickness of the upper SiO_2_ cladding layer. If we assume that the temperature of the Si layer under a buried oxide (BOX) layer in an SOI substrate is kept at the value controlled by a thermoelectric temperature controller, the change in temperature at the MRR, Δ*T*_MRR_, is roughly estimated to be Δ*T*_MRR_ = Δ*T*_s_ *d*_box_/(*d* + *d*_box_) considering the temperature gradient in the SiO_2_ layers, where Δ*T*_s_ is the change in temperature at the surface of the upper SiO_2_ cladding layer, and *d* and *d*_box_ are the thicknesses of the upper SiO_2_ cladding and BOX layers, respectively. This means that the lower the value of *d* is, the higher the temperature sensitivity. In the proposed sensor, *d* is 700 nm and *d*_box_ is 3 μm, therefore, Δ*T*_MRR_/Δ*T*_s_ is calculated to be approximately 0.8, which means that the change in temperature at the MRR decreases by 20%.

## 3. Fabrication

MRR sensors were fabricated on an SOI substrate using a complementary metal-oxide-semiconductor (CMOS)-compatible process. The thick upper SiO_2_ cladding layer in the vicinity of the Si-MRR was removed, and a 700-nm-thick SiO_2_ layer was deposited as a spacer between the MRR waveguide and the WO_3_ layer by magnetron sputtering.

A Pt-WO_3_ layer was deposited by a sol-gel method [30]. A precursor solution of Pt/WO_3_ was prepared as follows. First, hydrogen hexachloroplatinate (IV) hexahydrate was dissolved in distilled water. The solution was dissolved in ethanol aq. Then, 1 vol% acetylene glycol surfactant was added dropwise and the mixture was stirred for more than 30 min (Solution A). Next, 0.5 M sodium tungstate aq. was prepared by dissolving sodium tungstate (IV) dehydrate in distilled water. To gain 0.5 M tungstic acid aq., it was ion-exchanged with a hydrogen ion type of cation exchange resin. Then, tungsten acid was dissolved in Solution A and stirred. The Si-MRR sample was spin-coated with the precursor solution. The spin rate was 500 rpm and the spin time was 300 s. The sample was desiccated in a desiccator for 60 min and subsequently calcined in a furnace for 60 min at 500 °C. After calcination, it was cooled to room temperature in the furnace. The fabrication process is described in more detail in Ref. [30].

**Figure 3:**
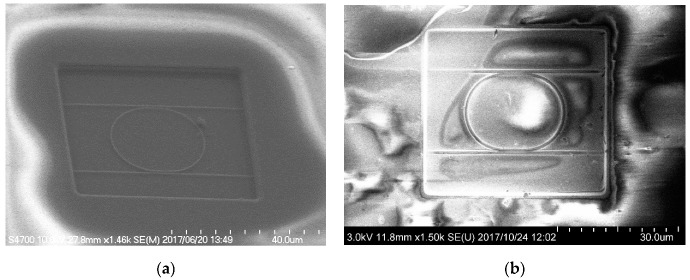
Scanning electron microscopy images of fabricated MRR (**a**) before deposition of Pt-WO_3_/SiO_2_ upper cladding layer and (**b**) after deposition of upper WO_3_/SiO_2_ layer [30].

**Figure 4:**
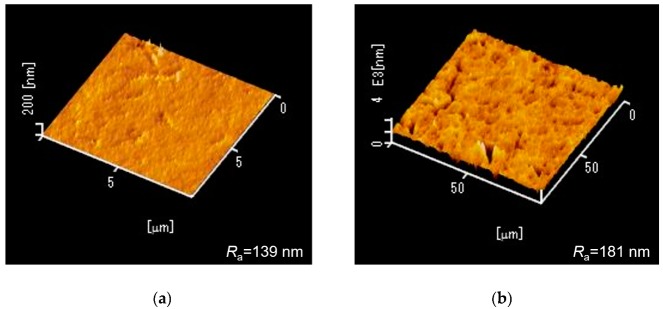
Atomic force microscopy images of the surface of the WO_3_ layer over areas of (**a**) 10 × 10 μm^2^ and (**b**) 100 × 100 μm^2^. *R*_a_ denotes average roughness.

**Figure 5:**
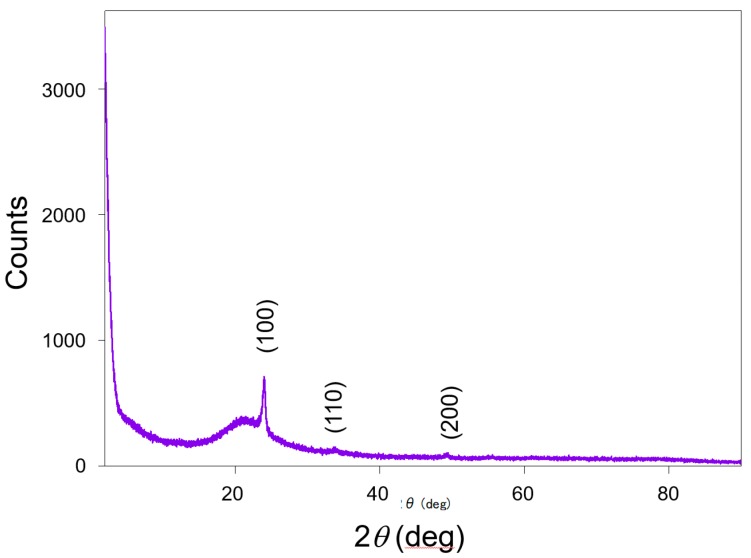
X-ray diffraction pattern of the WO_3_ layer.

Scanning electron microscopy (SEM) images of the fabricated MRR before the deposition of the Pt-WO_3_/SiO_2_ upper cladding layer and after the deposition of the WO_3_/SiO_2_ layer are shown in Figure 3a,b, respectively. The surface of the WO_3_ layer was macroscopically smooth. Figure 4 shows atomic force microscopy (AFM) images of the surface of the WO_3_ layer. There is some roughness of approximately 140–180 nm and some cracks were observed. This roughness and cracks increase the surface area of the WO_3_ layer, resulting in high sensitivity to hydrogen gas. Figure 5 shows an X-ray diffraction (XRD) pattern of the WO_3_ layer. Some peaks from the plane orientation (110) and other orientations were observed. These experimental results show that the WO_3_ layer is mainly amorphous but crystalline to some extent.

**Figure 6:**
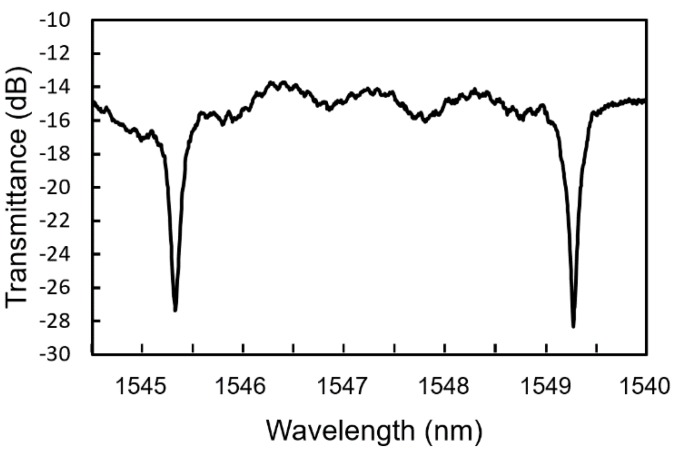
Transmittance spectrum of fabricated Si-MRR.

Figure 6 shows a transmittance spectrum of the fabricated MRR. The free spectral range (FSR) was approximately 4.05 nm, which is almost equal to the theoretical value. The Q factor of the MRR evaluated from the full width at half maximum of the resonant dips was 5164.

## 4. Sensing Characteristics of MRR Sensor

**Figure 7:**
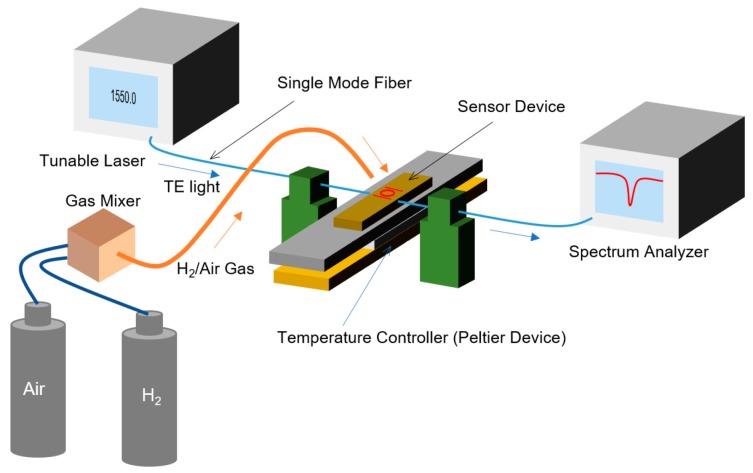
Schematic measurement setup for sensing characteristics of the MRR sensor.

Figure 7 shows a measurement setup for the characterization of the MRR sensor. The fabricated MRR sensor was directly exposed to hydrogen/air mixed gases with different hydrogen concentrations. Pure hydrogen and dry air from gas cylinders were mixed with a gas mixer (KOFLOC Corp., Kyoto, Japan). The flow rate of the mixed gas was fixed at 1.0 L/min with a mass flow controller in the gas mixer. The polarization of a continuous-wave light from a tunable laser (8164A, Agilent Technologies, Inc., Santa Clara, USA) was controlled to the transverse electric (TE) mode using a polarization controller (PAT9000B, Thorlabs Japan Inc., Tokyo, Japan), and the TE-polarized light was incident on the input port of the MRR sensor. The transmittance of the laser light in the vicinity of 1550 nm from the input port to the drop port was measured using a spectrum analyzer (Agilent, 8164A). The temperature of the sensor, *T*_sen_, was controlled using a thermoelectric temperature controller based on a Peltier device.

**Figure 8:**
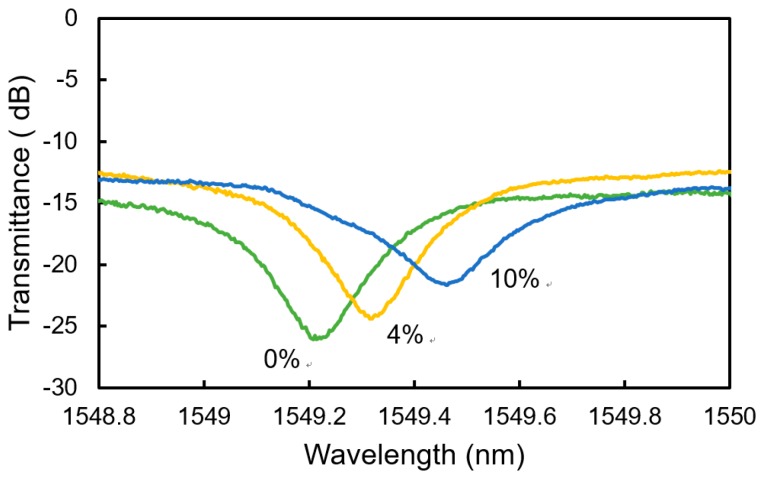
Measured spectra of the transmitted light for H_2_ concentration of 0 to 10 vol% at *T*_sen_ = 20 °C.

Figure 8 shows the measured spectra of the transmitted light for hydrogen gases with the concentrations of 0 to 10 vol% at *T*_sen_ = 20 °C. With the exposure to the gas, a resonant wavelength showed a redshift with a change in the depth of the dip at the resonant wavelength. The change in the depth of the dip was caused by the increase in the absorption coefficient when the WO_3_ formed H_x_WO_3_, which is discussed later.

**Figure 9:**
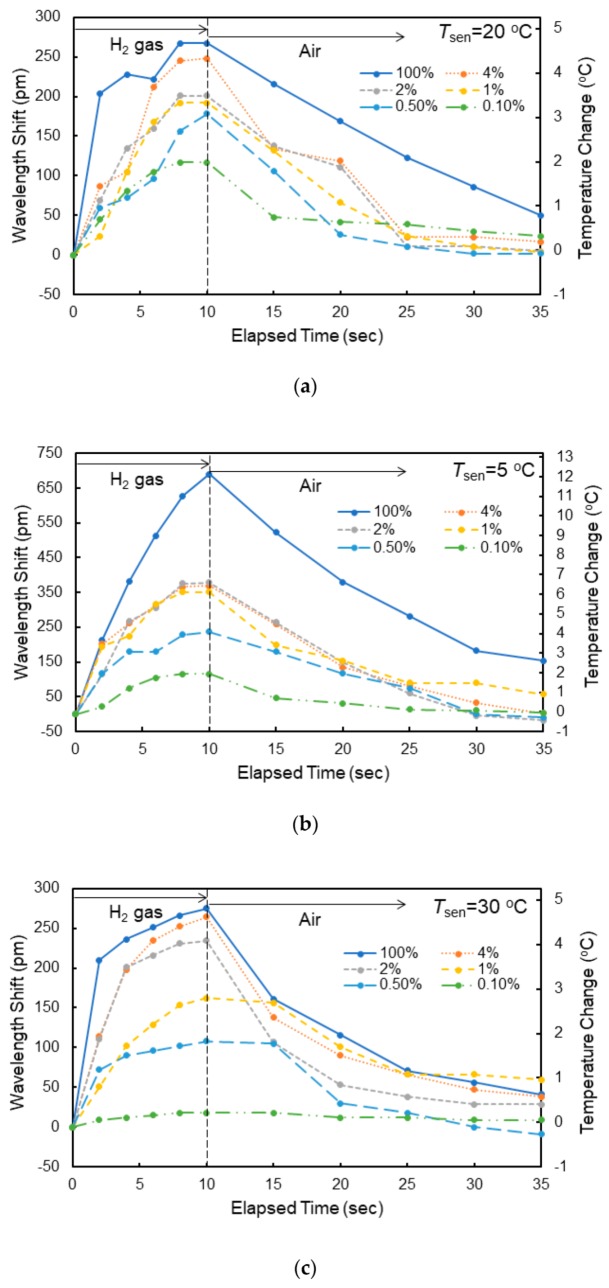
Resonant wavelength shift of MRR sensor when exposed to hydrogen/air mixed gases with different hydrogen concentrations measured at (**a**) 20 °C, (**b**) 5 °C, and (**c**) 30 °C.

**Figure 10:**
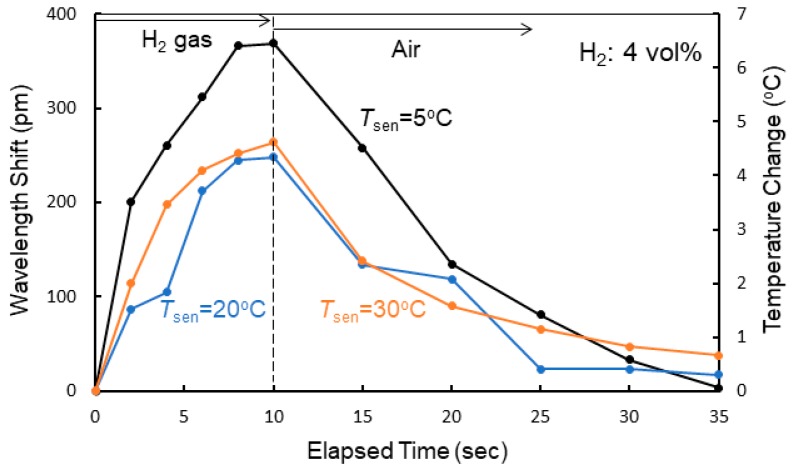
Wavelength shift as a function of elapsed time upon exposure to 4.0 vol% hydrogen gas at different temperatures.

Figure 9a shows the dependence of the shift in resonant wavelength on the elapsed time measured at *T*_sen_ = 20 °C for various hydrogen concentrations. The corresponding increases in temperature in the Si waveguide evaluated using the measured temperature coefficient d*λ*_0_/d*T* are also shown. The shift in resonant wavelength saturated at the elapsed time of 10 s after exposure to the hydrogen gas; therefore, the gas flow was stopped at 10 s in the experiment, and the atmosphere was switched to air. The response time was approximately 8 s for all gases. After stopping the exposure to the hydrogen gas, the resonant wavelength returned to its original value with the reaction of oxygen in air. Although the wavelength shift decreased with decreasing hydrogen gas concentration, a wavelength shift of as large as 117 pm was obtained for 0.1% hydrogen gas, showing the high sensitivity of the fabricated sensor to hydrogen gas. The shift in wavelength and response time depend on the gas flow rate. For example, the wavelength shift decreased by 20% and the response time increased by 50% for 4 vol% hydrogen gas at *T*_sen_ = 20 °C when the gas flow rate was changed from 1 to 0.25 L/min. This is probably because the rate at which hydrogen was absorbed into the WO_3_ layer decreased by lowering the gas flow rate. Figure 9b,c, respectively, shows the dependence of the shift in resonant wavelength on the elapsed time measured at *T*_sen_ = 5 and 30 °C for various hydrogen concentrations. At each temperature, similar resonant wavelength shifts were observed, but there was a difference in the magnitude of the wavelength shift. Figure 10 shows the wavelength shift as a function of the elapsed time upon exposure to 4.0 vol% hydrogen gas at different temperatures. The wavelength shift decreased with increasing *T*_sen_, although there was little difference in response time. There is a reading error of ± 5 pm and the resolution of a wavelength of the spectrum analyzer is 10 pm. Therefore, the accuracy of the wavelength is ± 15 pm (7.5% error for the wavelength shift of 200 pm) in the wavelength measurements.

**Figure 11:**
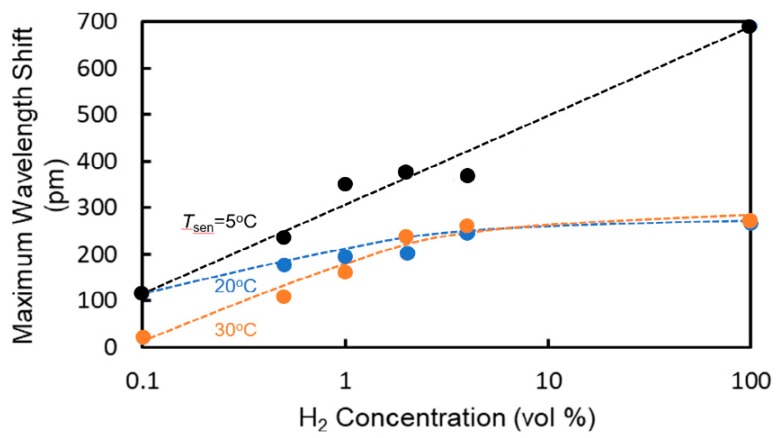
Dependence of wavelength shift at an elapsed time of 10 s on hydrogen concentration.

Figure 11 shows the dependence of the wavelength shift at an elapsed time of 10 s on the hydrogen concentration at each temperature. Although the wavelength shift shows dependence on *T*_sen_, high sensitivity to hydrogen was obtained at each temperature. The wavelength shifts saturated above 1 vol%. The cause of this saturation is considered to be the saturation of coverage of hydrogen molecules on the platinum catalyst, that is, the number of hydrogen molecules absorbed on the Pt-doped WO_3_ saturated. To increase the sensitivity at higher hydrogen concentrations, the dispersion of Pt atoms in the WO_3_ layer must be improved.

**Figure 12:**
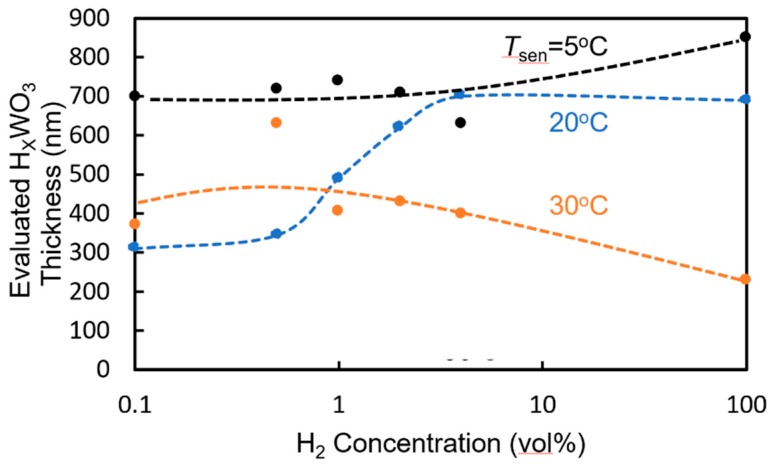
Relationship between the evaluated thickness of the H_x_WO_3_ layer on the surface side and hydrogen concentration of gas to which the sensor is exposed.

As shown in Figure 8, the depth of the dip at the resonant wavelength changes upon exposure to hydrogen gas. This change in dip depth is mainly caused by the increase in the absorption coefficient when WO_3_ forms H_x_WO_3_. However, the measured change is not consistent with that when the entire WO_3_ layer is assumed to form H_x_WO_3_. This shows that only part of the WO_3_ layer on the surface side forms H_x_WO_3_. We evaluated the thickness of the H_x_WO_3_ layer using the measured change in dip depth on the assumption that the cause of the change is the increase in the propagation loss of the WO_3_/H_x_WO_3_ layer. Figure 12 shows the relationship between the evaluated thickness of the H_x_WO_3_ layer on the surface side and the hydrogen concentration of the gas to which the sensor is exposed. At *T*_sen_ = 20 °C, the evaluated thickness of the H_x_WO_3_ layer increases with increasing hydrogen concentration. On the other hand, at *T*_sen_ = 5 °C, the evaluated thickness of the H_x_WO_3_ layer is approximately 700 nm, and the entire WO_3_ layer is considered to have formed H_x_WO_3_. In the case of *T*_sen_ = 30 °C, the evaluated thickness of the H_x_WO_3_ layer is approximately 400 nm and almost constant. One of the reasons for the dependence of the change in resonant wavelength on *T*_sen_ in Figure 9 is considered to be the change in the thickness of the H_x_WO_3_ layer with *T*_sen_. To improve the sensitivity to lower hydrogen concentrations of less than 2.0 vol% at higher *T*_sen_, the thickness of the WO_3_ layer must be decreased, even though the accuracy of hydrogen concentration measurement decreases at higher concentrations.

## 5. Design of MRR-Enhanced Mach–Zehnder Interferometer Sensor

**Figure 13:**
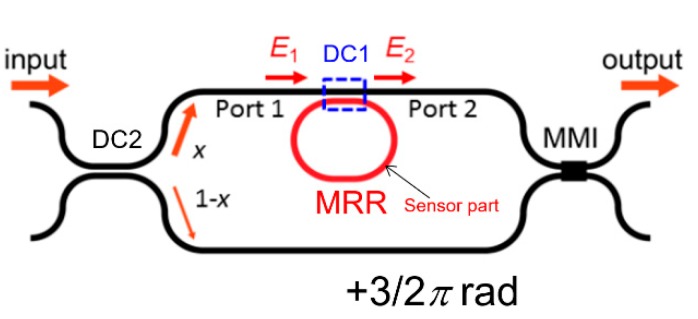
Schematic top view of the silicon-MRR-enhanced Mach–Zehnder interferometer (Si-MRR-MZI) sensor.

Although we discussed the Si-MRR gas sensor in the previous sections, an MRR-enhanced Mach–Zehnder interferometer (MRR-MZI) [32,33,34] enables us to detect hydrogen with much higher sensitivity. Figure 13 shows a schematic top view of the Si-MRR-MZI sensor. An all-pass-type MRR is coupled with one of the MZI arm waveguides through a directional coupler (DC1). The length of the arm waveguide without the MRR is designed to be greater than that of the other one by 3/2 *π* rad. The MRR acts as a sensor for hydrogen gas and its structure is assumed to be the same as that discussed in the previous sections. A directional coupler (DC2) with a light power splitting ratio of *x*:1−*x* is used as the input coupler to balance the light powers in both arms and obtain the highest extinction ratio. For *K* = 0.2, *x* is set to 0.572. For the output coupler, a 1 × 2 multimode interference (MMI) device is used.

**Figure 14:**
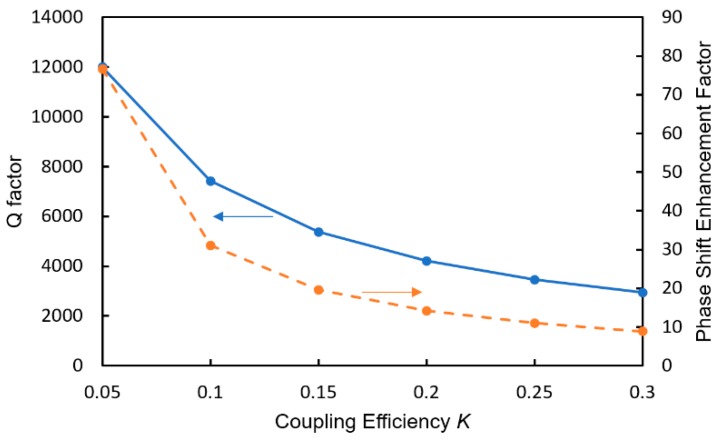
Dependence of the Q-factor and the phase shift enhancement factor Δ*φ*_eff_/Δ*φ* on *K*.

The MRR is a phaseshifter of the MZI and its phase shift is enhanced owing to the resonant effect in the MRR [32,33]. The phase shift enhancement factor is strongly dependent on the Q-factor of the MRR, and the Q-factor is determined by the power coupling efficiency *K* between the MRR and the arm waveguide. Figure 14 shows the dependence of the Q-factor and the phase shift enhancement factor Δ*φ*_eff_/Δ*φ* on *K*, where *φ*_eff_ and *φ* are the effective phase shift in the MRR and a single-pass phase shift, respectively.

**Figure 15:**
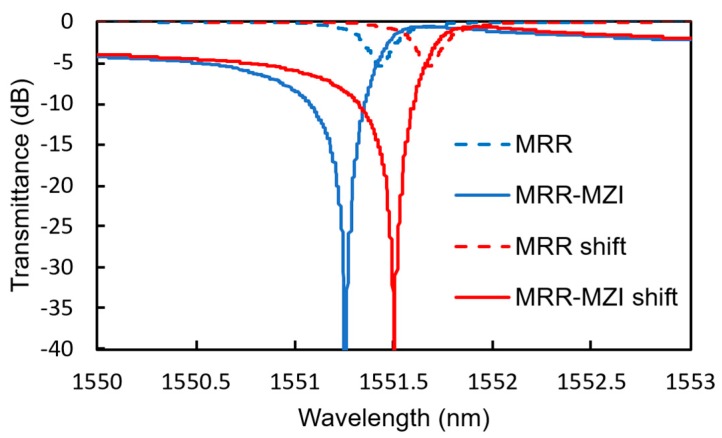
Transmittance spectra of the MRR-MZI with *K* = 0.2 (Q-factor = 4220).

Figure 15 shows the calculated transmittance spectra of an MRR-MZI and an MRR with *K* = 0.2 (Q-factor = 4220) before and after *T*_sen_ is changed by 4.4 °C, which corresponds to the change in temperature when 4.0 vol% hydrogen gas is detected. The light propagation loss in the waveguide was assumed to be 12 dB/cm. The power splitting ratio of the input MMI coupler is adjusted to obtain the highest extinction ratio. Owing to the interference effect of the MZI, a significantly high extinction ratio is obtained compared with that of the simple MRR, even though the shift in the resonant wavelength of the MRR-MZI is almost the same as that of the simple MRR.

**Figure 16:**
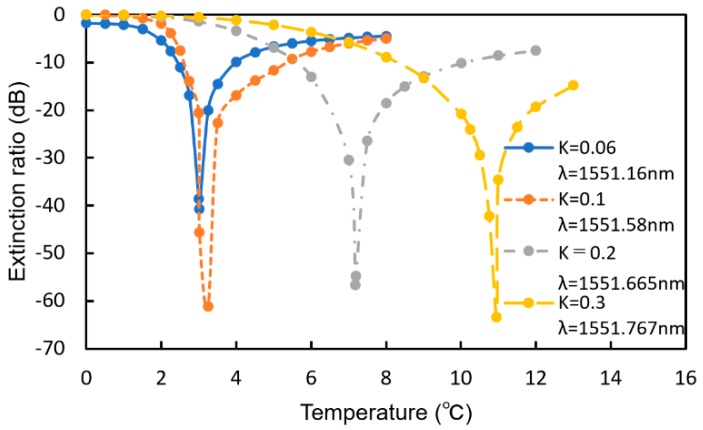
Extinction ratio as a function of the increase in temperature for various *K*.

Figure 16 shows the extinction ratio as a function of the increase in temperature for various coupling efficiencies (*K*). With decreasing *K*, the sensitivity to the temperature increases. If we assume that the MRR is the same as that previously discussed (*K* = 0.2, Q-factor = 4220), the temperature of the MRR increases by approximately 2.0 °C for 0.1 vol% hydrogen gas at *T*_sen_ = 20 °C, and a change in the extinction ratio larger than 40 dB is obtained if the operation wavelength is set at a suitable value, which is much larger than that of the simple MRR (less than 10 dB). One of the advantages of the MMR-MZI sensor is that the extinction ratio is very sensitive to the concentration of hydrogen. If the detecting wavelength is fixed at an appropriate value, the transmittance changes significantly upon exposure to hydrogen, and a much higher sensitivity can be realized without a spectrum analyzer.

## 6. Conclusions

We fabricated an optical hydrogen sensor based on a silicon MRR with a WO_3_/SiO_2_ layer using a CMOS-compatible process for the MRR and a sol-gel method for the WO_3_ layer and investigated its sensing characteristics at device temperatures of 5, 20, and 30 °C. At each temperature, a hydrogen concentration of as low as 0.1% was successfully detected with a response time of 10 s. The gas sensitivity increased with decreasing temperature. The dependence of the sensitivity on the device temperature can be attributed to the thickness of the H_x_WO_3_ layer formed by WO_3_ during exposure to hydrogen. In addition, a hydrogen gas sensor based on an Si-MRR-enhanced MZI was proposed and its significantly high sensing ability using improved changes in the transmittance of light was theoretically discussed. Such WO_3_/Si-MRR-based sensors are promising for simple but highly sensitive hydrogen gas sensing.

## Figures and Tables

**Table 1:** Device parameters of MRR sensor.

Symbol	Description	Value
*L*_MRR_	Round-trip length of MRR	69.3 μm
*L*_C_	Length of directional coupler	5.4 μm
*K*	Power coupling efficiency of directional coupler	0.2
*t*_WO3_	Thickness of WO_3_ upper cladding layer	700 nm
*t*_SiO2_	Thickness of SiO_2_ upper cladding layer	700 nm

**Table 2:** Refractive indices and extinction coefficients of tungsten oxide (WO_3_) and tungsten bronze (H_x_WO_3_).

Symbol	Description	WO_3_	H_x_WO_3_	Reference
*n*	Refractive index	2.0	2.4	[15]
*κ*	Extinction coefficient	0.1–0.3	1.0	[31]
